# An updated analysis of the epidemiologic trends of neuroendocrine tumors in Taiwan

**DOI:** 10.1038/s41598-021-86839-2

**Published:** 2021-04-12

**Authors:** Jeffrey S. Chang, Li-Tzong Chen, Yan-Shen Shan, Pei-Yi Chu, Chia-Rong Tsai, Hui-Jen Tsai

**Affiliations:** 1 National Institute of Cancer Research, National Health Research Institutes, 1F No 367, Sheng-Li Road, Tainan, 70456 Taiwan; 2 Department of Oncology, National Cheng Kung University Hospital, College of Medicine, National Cheng Kung University, Tainan, Taiwan; 3 Department of Internal Medicine, Kaohsiung Medical University Hospital, Kaohsiung, Taiwan; 4 Department of Surgery, National Cheng Kung University Hospital, College of Medicine, National Cheng Kung University, Tainan, Taiwan; 5 Institute of Clinical Medicine, National Cheng Kung University, Tainan, Taiwan; 6 Department of Pathology, Show Chwan Memorial Hospital, Changhua, Taiwan; 7 School of Medicine, College of Medicine, Fu Jen Catholic University, New Taipei City, Taiwan

**Keywords:** Neuroendocrine diseases, Cancer epidemiology

## Abstract

The incidence of neuroendocrine tumors (NETs) has been increasing in recent decades. Previously, we reported the incidence and survival of NETs in Taiwan by analyzing the 1996–2008 data from the Taiwan Cancer Registry. Here we performed an updated analysis on the incidence and survival of NETs in Taiwan from 1996 to 2015. The incidence of NETs was 0.244 per 100,000 in 1996 and increased to 3.162 per 100,000 in 2015. The most common site of NETs was rectum (29.65%), followed by lung/bronchus (17.22%), and pancreas (10.71%). The 5- and 10-year overall survival rates of all NETs were 54.6% and 45.3%, respectively. Female and younger NETs patients had a better survival. The survival of all NETs diagnosed between 2010 and 2015 was better than those diagnosed between 2004 and 2009. Among the common sites of NETs, an improved survival of pancreatic NETs diagnosed between 2010 and 2015 compared to those diagnosed between 2004 and 2009 was observed. Overall, the incidence of NETs in Taiwan has continued to increase. The survival of pancreatic NET has shown a recent improvement. The development of novel therapeutic agents has the potential to improve the prognosis of NETs of other sites in the near future.

## Introduction

Neuroendocrine tumors (NETs) are relatively rare neoplasms. These tumors can develop anywhere in the body and are heterogeneous in the classification based on the tumor grade and morphology. NETs of gastroentero-pancreatic (GEP) origin account for 50–60% of all NETs^1–3^. Rising incidence trends of NETs have been reported in many places around the world^1–3^. Dasari et al. reported a more rapid increase in the incidence of NETs in the recent decade^4^. The rising incidence trend of NETs is partly due to the increased awareness of NETs by the physicians and the execution of screening programs for prevalent cancers, particularly colorectal cancer.

Dasari et al. have shown an improved survival of GEP NETs in the US in this decade^4^. The improved prognosis of GEP NETs might be due to the development of the various novel agents and treatment modalities. Somatostatin analogues, such as long-acting octreotide, lanreotide and pasierotide, have been shown effective for treating GEP-NETs by phase II or III studies^5–8^. Everolimus and sunitinib have also been approved for the treatment of GEP-NETs and pulmonary NETs to prolong the progression free survival^9–11^. Peptide receptor radionuclide therapy (PRRT) has also been shown effective for treating advanced GEP and bronchial NETs^12,13^.

Previously, we reported the incidence and survival of NETs in Taiwan by analyzing the 1996–2008 data from the Taiwan Cancer Registry (TCR)^3^. We showed that the incidence of NETs in Taiwan was lower than that in the US and Europe and the common sites of NETs were different by race or ethnic group^1–3^. In this study, we updated our previous analysis and investigated the incidence and survival trends of NETs in Taiwan from 1996 to 2015.

## Results

### The trends of incidence of neuroendocrine tumors in Taiwan

**Figure 1:**
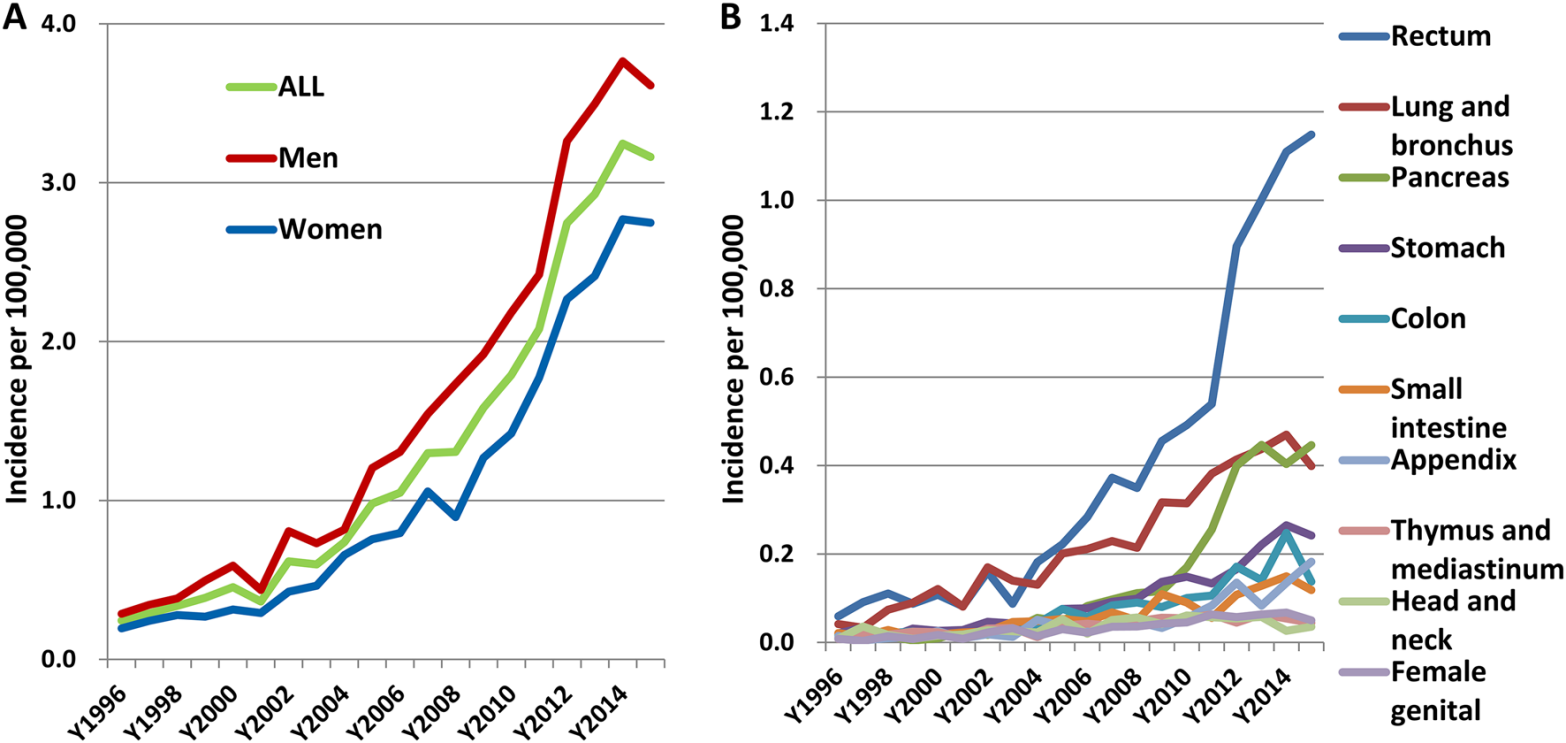
The annual incidence of NETs in Taiwan from 1996 to 2015. (**A**) The annual incidence of NETs of all sites in men, women and both. (**B**) The annual incidence of NETs in the 10 most common sites of NETs. Age-standardized incidence rates were calculated by direct standardization to the age distribution of the 2000 WHO standard population.

A total of 7760 cases of patients were diagnosed with neuroendocrine tumors (NETs) from 1996 to 2015 in Taiwan. Among these patients, 58.8% were men and 41.2% were women. The most common site of NETs was rectum (29.65%) followed by lung and bronchus (17.22%), pancreas (10.71%), stomach (7.69%), colon (5.79%), and small intestine (4.72%). Table 1 lists the numbers and percentages of NETs by site. To analyze the incidence trend of NETs, we divided the time of diagnosis into three time periods: T1 (1996–2003), T2 (2004–2009), and T3 (2010–2015). The percentages of rectal and pancreatic NETs showed an increasing trend from T1 to T2 to T3. The percentage of rectal NET was 23.99% in T1 and increased to 26.67% and 31.64% in T2 and T3, respectively. The percentage of pancreatic NET was 3.65% in T1 and increased to 7.27% and 13.09% in T2 and T3, respectively. The incidence of NETs increased overall and in both men and women with a more rapid rising trend in the later years (Fig. 1A and Supplementary Table S1). The incidence of all NETs increased from 0.244 per 100,000 in 1996 to 3.162 per 100,000 in 2015 [annual percentage change (APC) = 15.44, *P* < 0.0001], while for men, the incidence increased from 0.287 per 100,000 in 1996 to 3.612 per 100,000 in 2015 (APC = 15.26, *P* < 0.0001) and for women, the incidence increased from 0.196 per 100,000 in 1996 to 2.748 per 100,000 in 2015 (APC = 15.99, *P* < 0.0001). The different sites of NETs all showed an increasing incidence trend but with different rates of increase (Supplementary Table S1). Figure 1B shows the annual incidence of NETs in the 10 most common sites. The incidence of rectal NET was 0.059 per 100,000 in 1996 and rapidly increased to 1.149 per 100,000 in 2015 with an APC of 17.34 (*P* < 0.0001). The incidence of NET in lung and bronchus was 0.042 per 100,000 in 1996 and rose to 0.399 per 100,000 in 2015 with an APC of 13.42 (*P* < 0.0001). The incidence of pancreatic NET increased from 0.017 per 100,000 in 1996 to 0.446 per 100,000 in 2015 with an APC of 28.04 (*P* < 0.0001).

**Table 1:** The number and percentage of NETs by site from 1996 to 2015.

	1996–2015 N = 7760	T1 (1996–2003) N = 767	T2 (2004–2009) N = 1927	T3 (2010–2015) N = 5066
	N (%)	N (%)	N (%)	N (%)
Rectum	2301 (29.65)	184 (23.99)	514 (26.67)	1603 (31.64)
Lung and bronchus	1336 (17.22)	173 (22.56)	364 (18.89)	799 (15.77)
Pancreas	831 (10.71)	28 (3.65)	140 (7.27)	663 (13.09)
Stomach	597 (10.71)	53 (6.91)	149 (7.73)	395 (7.8)
Colon	449 (5.79)	38 (4.95)	119 (6.18)	292 (5.76)
Small intestine	366 (4.72)	43 (5.61)	105 (5.45)	218 (4.3)
Appendix	275 (3.54)	25 (3.26)	64 (3.32)	186 (3.67)
Thymus and mediastinum	207 (2.67)	41 (5.35)	64 (3.32)	102 (2.01)
Head and neck	194 (2.5)	37 (4.82)	66 (3.43)	91 (1.8)
Female genital	189 (2.44)	28 (3.65)	50 (2.59)	111 (2.19)
Breast	142 (1.83)	1 (0.13)	37 (1.92)	104 (2.05)
Liver	104 (1.34)	15 (1.96)	30 (1.56)	59 (1.16)
Esophagus	81 (1.04)	5 (0.65)	23 (1.19)	53 (1.05)
Female gonads	66 (0.85)	8 (1.04)	17 (0.88)	41 (0.81)
Urinary tract	65 (0.84)	4 (0.52)	21 (1.09)	40 (0.79)
Biliary tract	53 (0.68)	4 (0.52)	12 (0.62)	37 (0.73)
Prostate	40 (0.52)	3 (0.39)	11 (0.57)	26 (0.51)
Others	464 (5.98)	77 (10.04)	141 (7.32)	246 (4.86)

### The distribution of the morphologic types of NETs in Taiwan

The distribution of the morphologic types of NETs in the 6 most common sites is shown in Table 2. The distribution of the morphologic type of NETs is variable among different sites. Because the grade of NETs is not available in the TCR, we analyzed the morphologic types of NETs using the M codes and classified NETs into carcinoid (8240), atypical carcinoid (8249), neuroendocrine carcinoma (8246), large cell neuroendocrine carcinoma (8013), and others. From 1996 to 2015, the percentage of carcinoid was higher in NETs of rectum (83.6%) and small intestine (61.7%) but lower in NETs of lung and bronchus (25.8%) and pancreas (29.1%). The proportions of morphological types in NETs of the 6 common sites varied in men and women and in the three time periods (Supplementary Table S2). Particularly, the proportion of carcinoid in NETs of lung and bronchus decreased from T1 (44.5%) to T2 (23.1%) and T3 (19.3%). In contrast, the proportion of large cell neuroendocrine carcinoma increased from T1 (13.9%) to T2 (30.2%) and T3 (34.2%). The percentage of carcinoid in gastric NETs also decreased from 73.6% in T1 to 45.6% in T2 and 39.7% in T3. However, the percentage of carcinoid remained persistently higher in women than that in men in all three time periods.

**Table 2:** The number and percentage of NETs in the 6 most common sites by morphological type.

	Rectum	Lung and bronchus	Pancreas	Stomach	Colon	Small intestine
	N (%)	N (%)	N (%)	N (%)	N (%)	N (%)
**Morphological type (1996–2015)**						
Carcinoid (8240)	1923 (83.6)	315 (23.6)	242 (29.1)	264 (44.2)	185 (41.2)	226 (61.7)
Atypical carcinoid (8249)	–	99 (7.4)	–	–	–	–
Neuroendocrine carcinoma (8246)	240 (10.4)	418 (31.3)	417 (50.2)	169 (28.3)	110 (24.5)	99 (27)
Large cell neuroendocrine carcinoma (8013)	–	407 (30.5)	–	–	–	–
Others	138 (6)	97 (7.3)	172 (20.7)	164 (27.5)	154 (34.3)	41 (11.2)

### The survival of NETs in Taiwan

**Figure 2:**
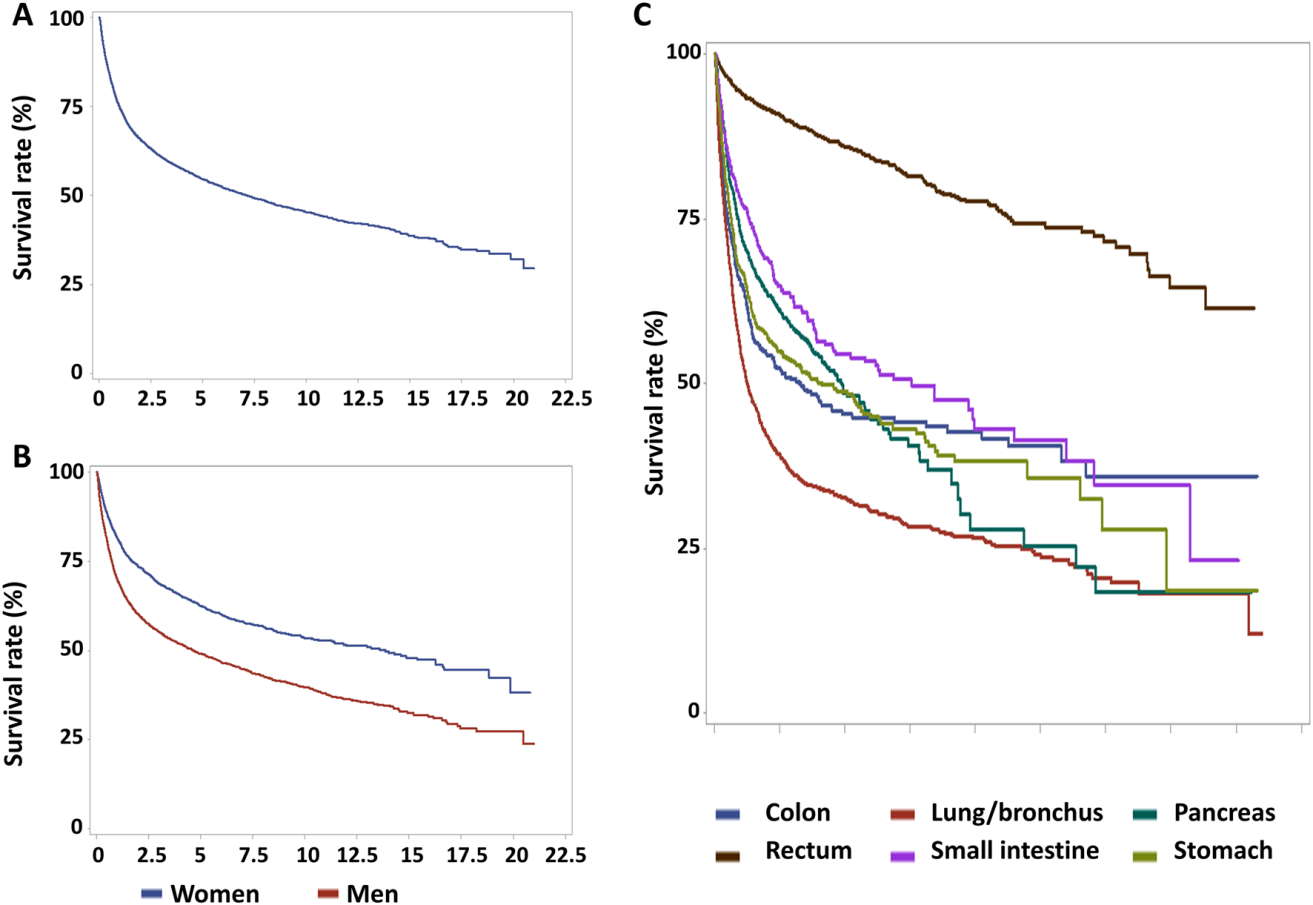
The overall survival of NETs. (**A**) The overall survival of NETs for all patients (**B**) The overall survival of NETs by sex. (**C**) The overall survival of NETs in the 6 most common sites.

The results for the overall survival of NETs for all patients and by sex are shown in Fig. 2A,B. The 1-, 3-, 5-, and 10-year survival rates of all NETs and by site and sex from 1996 to 2015 are shown in Table 3. The 1-, 3-, 5-, and 10-year overall survival rates for all NETs from 1996 to 2015 were 74.5%, 60.8%, 54.6%, and 45.3%, respectively. The overall survival curves of NETs in the 6 most common sites are shown in Fig. 2C. Among these 6 sites, the best survival was observed in NETs of rectum and small intestine and the worst survival occurred in NETs of lung. However, when all sites were considered, better survival was also observed in NETs of appendix and breast and worse survival was observed in NETs of esophagus, liver and biliary tract (Table 3). To analyze the prognostic factors associated with the survival of NETs, we performed Cox proportional hazards regression analysis by primary site, sex, age and the time periods of diagnosis (Table 4). Compared to the survival of rectal NETs, NETs in other sites all had a worse survival, with NETs of liver and esophagus showing the worst survival (liver: HR 8.71; 95% CI 6.89–11.01; esophagus: HR 8.69; 95% CI 6.71–11.27) according to the multivariate analysis. Women had a better survival than men (HR 0.64; 95% CI 0.59–0.69) according to the multivariate analysis. A worse survival was observed with increasing age. Compared to the survival of NETs diagnosed in T1 (1996–2003), the survival was worse in T2 (HR 1.17; 95% CI 1.05–1.31) and not significantly different in T3 (HR 1.08; 85% CI 0.97–1.2) according to the multivariate analysis.

**Table 3:** The 1-, 3-, 5-, and 10-year overall survival rates of NETs by sites and sex in Taiwan (1996–2015).

	1-year			3-year			5-year			10-year		
	All	Men	Women	All	Men	Women	All	Men	Women	All	Men	Women
All	0.745	0.696	0.814	0.608	0.552	0.688	0.546	0.490	0.626	0.453	0.397	0.535
**Primary site**												
Rectum	0.942	0.932	0.956	0.897	0.886	0.912	0.860	0.845	0.883	0.775	0.763	0.795
Lung and bronchus	0.545	0.454	0.769	0.364	0.253	0.638	0.326	0.220	0.591	0.264	0.172	0.496
Pancreas	0.728	0.690	0.767	0.583	0.546	0.621	0.488	0.438	0.542	0.283	0.265	0.303
Stomach	0.670	0.577	0.813	0.532	0.415	0.714	0.487	0.378	0.655	0.378	0.273	0.549
Colon	0.650	0.654	0.645	0.505	0.513	0.494	0.452	0.465	0.436	0.426	0.433	0.418
Small intestine	0.779	0.784	0.770	0.629	0.593	0.690	0.544	0.485	0.644	0.434	0.361	0.566
Appendix	0.931	0.942	0.917	0.826	0.850	0.796	0.762	0.784	0.733	0.660	0.620	0.733
Thymus and mediastinum	0.787	0.750	0.902	0.558	0.547	0.590	0.403	0.402	0.409	0.222	0.218	0.230
Head and neck	0.753	0.716	0.849	0.507	0.468	0.613	0.380	0.345	0.475	0.275	0.231	0.390
Female genital	0.693	–	0.693	0.438	–	0.438	0.358	–	0.358	0.324	–	0.324
Breast	0.951	1.000	0.950	0.819	1.000	0.818	0.730	1.000	0.727	0.571	1.000	0.558
Liver	0.490	0.391	0.650	0.301	0.234	0.406	0.162	0.085	0.279	0.081	0.032	0.155
Esophagus	0.444	0.440	0.500	0.141	0.140	0.167	0.092	0.084	0.167			
Female gonads	0.833	–	0.833	0.657	–	0.657	0.571	–	0.571	0.571	–	0.571
Urinary tract	0.677	0.737	0.593	0.524	0.542	0.499	0.473	0.502	0.428	0.356	0.452	0.267
Biliary tract	0.604	0.722	0.543	0.284	0.368	0.243	0.216	0.368	0.130	0.216	0.368	
Prostate	0.725	0.725	–	0.445	0.445	–	0.377	0.377	–	0.118	0.118	–
Others	0.481	0.423	0.563	0.291	0.239	0.367	0.224	0.166	0.311	0.147	0.106	0.211

**Table 4:** Cox proportional hazards regression analysis for the survival of NETs.

	Univariate		Multivariate	
	HR	95% CI	HR	95% CI
**Primary site**				
**Referent: rectum**				
Lung and bronchus	7.21	6.37–8.16	5.29	4.66–6.0
Pancreas	4.3	3.72–4.98	4.13	3.57–4.78
Stomach	4.8	4.12–5.6	3.42	2.93–4.0
Colon	4.86	4.12–5.73	3.96	3.35–4.67
Small intestine	3.63	3.02–4.37	2.64	2.19–3.19
Appendix	1.61	1.23–2.1	1.5	1.15–1.95
Thymus and mediastinum	5.41	4.46–6.57	4.63	3.81–5.63
Head and neck	5.42	4.44–6.63	4.48	3.66–5.48
Female genital	5.58	4.54–6.87	7.68	6.2–9.51
Breast	1.69	1.19–2.41	1.67	1.17–2.39
Liver	10.44	8.26–13.18	8.71	6.89–11.01
Esophagus	11.87	9.17–15.36	8.69	6.71–11.27
Female gonads	2.81	1.89–4.19	3.85	2.58–5.76
Urinary tract	4.74	3.34–6.7	3.45	2.44–4.89
Biliary tract	8.04	5.75–11.24	5.67	4.05–7.95
Prostate	6.58	4.47–9.67	3.47	2.36–5.11
Others	9.59	8.27–11.13	7.72	6.65–8.98
**Sex**				
**Referent: men**				
Women	0.64	0.59–0.68	0.64	0.59–0.69
**Age**				
**Referent: age < 40**				
40–50	1.44	1.22–1.7	1.31	1.11–1.54
50–60	2.2	1.89–2.56	1.9	1.63–2.21
60–70	3.28	2.83–3.8	2.46	2.12–2.85
> 70	6.66	5.78–7.67	4.65	4.03–5.37
**Time period of diagnosis**				
**Referent: 1996–2003 (T1)**				
2004–2009 (T2)	1.02	0.92–1.14	1.17	1.05–1.31
2010–2015 (T3)	0.84	0.76–0.94	1.08	0.97–1.2

### The survival trends of NETs in the six most common sites in Taiwan

To analyze whether the survival of NETs in different site had improved in the recent two decades, we calculated the 1-, 3-, 5- and 10-year overall survival rates of NETs of the six most common sites diagnosed in the three time periods (T1, T2, and T3) (Supplementary Table S3). We performed the Cox proportional hazards regression analysis for the survival of NETs in the 6 common sites separately to examine the influence of age, sex and the time periods of diagnosis (T2 vs. T3) (Table 5). We only included data from T2 and T3 because the case number was much lower in T1 (767) than those in T2 (1927) and T3 (5066) and the diagnostic rate of NETs before 2003 was also much lower likely to the lower awareness of NETs by the physicians, thus, the case number in T1 was too small for the analysis stratified by the 6 sites of NETs. The results showed that the women generally had a better survival than men for the 6 common sites of NETs except for colonic NETs (HR 0.89, 95% CI 0.68–1.18 by multivariate analysis), the survival of which was not significantly different between men and women. Generally, NETs diagnosed at older age had worse survival than those diagnosed at younger age for all six common sites of NETs. Improved survival from T2 to T3 was only observed in pancreatic NETs (HR 0.52, 95% CI 0.41–0.66 by multivariate analysis) (Table 5).

**Table 5:** The Cox proportional hazards regression analysis for the survival of NETs in the 6 common sites.

Site of NETs	Rectum				Lung/Bronchus			
	Univariate		Multivariate		Univariate		Multivariate	
	HR	95% CI	HR	95% CI	HR	95% CI	HR	95% CI
**Sex**								
**Referent: Men**								
Women	0.76	0.59–0.97	0.78	0.61–1.00	0.64	0.59–0.68	0.39	0.32–0.46
**Age**								
**Referent: < 40**								
40 ≤ age < 50	1.47	0.79–2.76	1.49	0.79–2.78	2.02	1.29–3.17	1.93	1.17–3.20
50 ≤ age < 60	3.58	2.07–6.19	3.63	2.10–6.28	2.75	1.81–4.18	2.15	1.34–3.44
60 ≤ age < 70	5.65	3.25–9.80	5.67	3.27–9.85	3.2	2.13–4.81	2.37	1.49–3.76
70 ≤ age	20.73	12.3–35.1	20.5	12.1–34.7	6.57	4.41–9.80	5.07	3.22–7.98
**The time period of diagnosis**								
**Referent: T2 (2004–2009)**								
T3 (2010–2015)	0.77	0.59–1.01	0.86	0.65–1.13	1.08	0.94–1.23	1.02	0.88–1.18

Site of NETs	Pancreas				Stomach			
	Univariate		Multivariate		Univariate		Multivariate	
	HR	95% CI	HR	95% CI	HR	95% CI	HR	95% CI
**Sex**								
**Referent: men**								
Women	0.77	0.63–0.95	0.75	0.60–0.92	0.44	0.34–0.58	0.56	0.43–0.74
**Age**								
**Referent: < 40**								
40 ≤ age < 50	1.41	0.92–2.17	1.36	0.89–2.08	1.01	0.31–3.28	0.82	0.25–2.68
50 ≤ age < 60	1.62	1.09–2.41	1.59	1.07–2.37	2.28	0.80–6.47	2	0.70–5.70
60 ≤ age < 70	2.31	1.56–3.42	2.28	1.54–3.37	4.85	1.76–13.4	4.05	1.47–11.21
70 ≤ age	3.92	2.66–5.77	3.87	2.63–5.70	10.01	3.71–27.0	7.79	2.87–21.20
**The time period of diagnosis**								
**Referent: T2 (2004–2009)**								
T3 (2010–2015)	0.55	0.43–1.69	0.52	0.41–0.66	1	0.76–1.30	1.03	0.79–1.35

Site of NETs	Colon				Small intestine			
	Univariate		Multivariate		Univariate		Multivariate	
	HR	95% CI	HR	95% CI	HR	95% CI	HR	95% CI
**Sex**								
**Referent: men**								
Women	1.03	0.79–1.35	0.89	0.68–1.18	0.72	0.50–1.03	0.66	0.45–0.95
**Age**								
**Referent: < 40**								
40 ≤ age < 50	1.75	0.85–3.64	1.76	0.85–3.66	1.08	0.34–3.39	1.12	0.36–3.53
50 ≤ age < 60	2.66	1.34–5.26	2.66	1.34–5.27	1.81	0.63–5.20	1.84	0.64–5.30
60 ≤ age < 70	2.58	1.30–5.09	2.57	1.30–5.09	3.08	1.09–8.68	3	1.06–8.44
70 ≤ age	5.07	2.63–9.76	5.18	2.69–9.99	4.45	1.61–12.3	4.74	1.71–13.17
**The time period of diagnosis**								
**Referent: T2 (2004–2009)**								
T3 (2010–2015)	0.9	0.67–1.20	0.98	0.73–1.31	0.75	0.53–1.07	0.92	0.64–1.32

### The analysis for survival by the morphologic type of NETs and by sex for the six most common sites

We analyzed the 5-year overall survival rate of NETs in the 6 most common sites by morphologic type (M code) and by sex from 2010 to 2015 (Table 6). Generally, the 5-year survival rate was higher for the carcinoid morphologic type. The 5-year survival rates of carcinoids in rectum, lung/bronchus, pancreas, stomach, colon and small intestine were 95%, 84%, 85%, 79%, 87%, and 78%, respectively. The 5-year survival rate of atypical carcinoids in lung and bronchus was 64%. The 5-year survival rates of neuroendocrine carcinoma in rectum, lung/bronchus, pancreas, stomach, colon and small intestine were 63%, 12%, 32%, 26%, 27%, and 48%, respectively. The 5-year survival rate of large cell neuroendocrine carcinoma in lung/bronchus was 15%. Generally, women had higher survival rate than men for either carcinoids or neuroendocrine carcinoma in each site except for rectum and colon.

**Table 6:** The 5-year overall survival rate of NETs in the 6 most common sites.

Morphologic type	Lung and bronchus								
	1996–2003 (T1) (N = 173)			2004–2009 (T2) (N = 364)			2010–2015 (T3) (N = 799)		
	ALL	Men	Women	ALL	Men	Women	ALL	Men	Women
Carcinoid	0.6623	0.6	0.75	0.7738	0.7	0.8148	0.8442	0.7667	0.8898
Atypical carcinoid	0.4	–	0.6667	0.5161	0.5	0.5714	0.6401	0.5379	0.7104
Neuroendocrine carcinoma	0.1077	0.0714	0.3333	0.0984	0.0417	0.3077	0.1237	0.0815	0.2905
Large cell neuroendocrine carcinoma	0.3333	0.3333	0.3333	0.1545	0.14	0.3	0.1521	0.1355	0.2557
Others	0.5	0.5	–	0.0625	0.0625	–	0.1604	0.1997	–

Morphologic type	Rectum								
	1996–2003 (T1) (N = 184)			2004–2009 (T2) (N = 514)			2010–2015 (T3) (N = 1603)		
	ALL	Men	Women	ALL	Men	Women	ALL	Men	Women
Carcinoid	0.8324	0.7885	0.8696	0.9101	0.9049	0.9176	0.9457	0.9367	0.9594
Neuroendocrine carcinoma	0.2222	0.2	0.25	0.3958	0.303	0.6	0.6341	0.6263	0.656
Others	0.5	0.5	–	0.381	0.4286	0.2857	0.4415	0.3934	0.527

Morphologic type	Pancreas								
	1996–2003 (T1) (N = 28)			2004–2009 (T2) (N = 140)			2010–2015 (T3) (N = 663)		
	ALL	Men	Women	ALL	Men	Women	ALL	Men	Women
Carcinoid	0.5714	0.5	0.6667	0.1667	–	0.25	0.8489	0.8259	0.8674
Neuroendocrine carcinoma	0.4	0.1818	0.6667	0.3435	0.3208	0.359	0.3225	0.2845	0.3748
Others	–	–	–	–	–	–	0.5886	0.5689	0.6275

Morphologic type	Stomach								
	1996–2003 (T1) (N = 53)			2004–2009 (T2) (N = 149)			2010–2015 (T3) (N = 395)		
	ALL	Men	Women	ALL	Men	Women	ALL	Men	Women
Carcinoid	0.5128	0.3913	0.6875	0.7206	0.6452	0.7838	0.7911	0.7778	0.7941
Neuroendocrine carcinoma	0.2727	0.2	1	0.3469	0.3421	0.3636	0.2622	0.178	0.4256
Others	–	–	–	0.1875	0.1852	0.2	0.2951	0.2394	0.45875

Morphologic type	Colon								
	1996–2003 (T1) (N = 38)			2004–2009 (T2) (N = 119)			2010–2015 (T3) (N = 292)		
	ALL	Men	Women	ALL	Men	Women	ALL	Men	Women
Carcinoid	0.7	0.5882	0.8462	0.7143	0.75	0.6471	0.8654	0.8243	0.9395
Neuroendocrine carcinoma	–	–	–	0.2286	0.1905	0.2857	0.2682	0.2826	0.2909
Others	–	–	–	0.1714	0.1818	0.1538	0.2369	0.3104	0.1854

Morphologic type	Small intestine								
	1996–2003 (T1) (N = 43)			2004–2009 (T2) (N = 105)			2010–2015 (T3) (N = 218)		
	ALL	Men	Women	ALL	Men	Women	ALL	Men	Women
Carcinoid	0.5	0.4231	0.75	0.6613	0.5897	0.7826	0.7787	0.7356	0.837
Neuroendocrine carcinoma	0.4286	0.4	0.5	0.2432	0.2	0.3333	0.4845	0.4442	0.533
others	–	–	–	0.3333	0.2	1	0.0987	0.1048	0.0866

–: not available

## Discussion

In this study, we showed that the incidence of NETs increased from 0.244 per 100,000 in 1996 to 3.162 per 100,000 in 2015. Rectum, lung/bronchus, and pancreas were the most 3 common primary sites of NETs. The best survival of NETs was for NETs in rectum and small intestine and the worst survival was observed for NETs in esophagus and liver. During our study period, improved survival was only observed for pancreatic NETs.

Increasing incidence trends of NETs have been observed in many countries, including Taiwan. Several factors may have contributed to the increasing incidence trend of NETs in Taiwan. One possible reason is the increasing awareness of NETs by the physicians, particularly pathologists. Another possible explanation is the wide availability of diagnostic facilities in Taiwan, including endoscopy, sonography, and CT scan in clinics, regional hospitals and medical centers. Once a physician observes an abnormal finding, the physician will arrange additional examinations to make a final diagnosis. Additionally, cancer screening programs in Taiwan may have also contributed to the increased incidence, particular for NETs of gastrointestinal tract. National cancer screening program for cervical, breast, oral, and colon cancers in Taiwan began in 2010. People who have had a positive stool occult blood test would have received endoscopic examination for gastrointestinal tract, such as pan-endoscopy and/or colonoscopy. However, the incidence trends also increased for NETs located in sites other than the gastrointestinal tract, including NETs in sites not easily detectable through screening. In addition, if the increased physicians’ awareness of NETs is a major factor, one would expect the incidence of NETs to reach a plateau at some point, but our results indicated the contrary, showing a continued increase in NETs incidence. Taken together, our results suggest a real increase in the incidence of NETs in Taiwan.

Among the primary sites, the incidence of pancreatic NETs increased most rapidly. The result is consistent with the NET incidence in the US reported by Dasari et al.^4^. Our previous study found that although both pancreatic adenocarcinoma and pancreatic NETs showed increasing incidence, the increase in pancreatic NETs was more prominent, suggesting that the etiologies of adenocarcinoma and NETs might be partly different^14^. Halfdanarson et al. conducted a case–control study of 263 pancreatic NET patients and 602 controls and showed that non-alcohol use, diabetes, obesity, and family cancer history of certain cancers (sarcoma, pancreatic NETs, gall bladder cancer, ovarian cancer, and gastric cancer) were risk factors of pancreatic NETs^15^. Haugvik et al. performed a meta-analysis for risk factors of pancreatic NETs and reported that diabetes, family history of cancer, alcohol, and smoking were risk factors^16^. The other risk factors for NETs in sites other than pancreas include family history of carcinoid or other cancers, race, sex, age, alcohol drinking, social economic status, obesity, smoking, and LDL-cholesterol level^17–21^. However, more investigations are needed to generate solid evidence.

In our updated analysis (data from 1996 to 2015), we observed that since our last analysis (data from 1996 to 2008), rectum and lung and bronchus have the remained the most and the second most common site of NETs occurring in Taiwan. The incidence of pancreatic NETs surpassed the incidence of gastric NETs since our last analysis to rank third after rectal and lung and bronchus NETs. Our results revealed an abrupt increase of rectal NETs in 2011, which might be partly due the initiation of the national cancer screening program in 2010 targeting four cancers, including colon cancer. The incidence trends of NETs in other sites in Taiwan were similar to those in the US reported by Dasari et al.^4^.

The common sites of NETs are different by races and geographic areas^3^. NETs of lung and bronchus are common among white Americans, Europeans and Asians. NETs of rectum are more common among Asians and the African Americans whereas NETs of small intestine are more common among white Americans and Europeans. The site distribution of NETs in Oceania is also different. Wyld et al. reported the incidence trends of NETs over three decades in Queensland, Australia and showed that the most common sites of NETs were small intestine (1.03 per 100,000), followed by appendix (0.74 per 100,000), lung (0.71 per 100,000), and rectum (0.69 per 100,000). Pancreatic NETs (0.25 per 100,000) was the sixth most common NETs in their analysis^22^. Hallet et al. also reported increasing incidence trends of NETs in Ontario, Canada from 1994 to 2009. In that study, the most common sites of NETs in 2009 were lung/bronchus (1.28 per 100,000), followed by small intestine (1.01 per 100,000), rectum (0.96 per 100,000), large intestine (0.69 per 100,000), and pancreas (0.6 per 100,000)^23^. These studies showed that the most common sites of NETs are different by races and geographic areas, suggesting possible genetic and environmental differences in the etiologies of the NETs. Therefore, it would be crucial to survey the risk factors in different races and geographic areas to identify the potential genetic and environmental factors for the prevention of NETs.

In this study, we noticed that among the 6 common sites of NETs, only the survival of pancreatic NETs improved from T2 to T3. The survival of NETs in other sites showed no significant improvement. NETs of lung and bronchus had the worst survival among the NETs in the 6 most common sites with a 5-year overall survival rate of 32.6% (Table 3). There had been no specific novel agents approved for NETs in lung and bronchus until recently. Everolimus was approved to prolong the progression free survival of well differentiated pulmonary NETs due to the results of a successful phase III study^11^ and the National Health Insurance of Taiwan began reimbursing everolimus for treating pulmonary NETs in October, 2019. However, there is still limited improvement for the treatment of high grade neuroendocrine carcinoma, including large cell neuroendocrine carcinoma. The prognosis of pulmonary large cell neuroendocrine carcinoma was poor with a 5-year cancer-specific survival of 20.7% according to the analysis from the Surveillance, Epidemiology, and End-Results (SEER) database from 2000 to 2013^24^. The overall survival of the high grade pulmonary large cell neuroendocrine carcinoma patients with distant stage was not different from that of small cell lung cancer^25^. Most neuroendocrine carcinoma, including large cell neuroendocrine carcinoma, in lung or other sites have been treated as small cell lung cancer. Chemotherapy with etoposide and platinum is the standard first line treatment^26,27^. There is no standard second line treatment for high grade neuroendocrine carcinoma after recurrence or refractory. Although many novel agents, such as pembrolizumab, topoisomerase-1-inhibiting antibody–drug conjugate (ADC) targeting Trop-2 (sacituzumab govitecan), temozolomide, and antibody–drug conjugate directed against delta-like protein 3 (rovalpituzumab tesirine), have been tested for their toxicity and clinical efficacy in recurrence or resistant small cell lung cancer^28–31^, studies of novel agents in neuroendocrine carcinoma of other sites is limited. Further investigations are needed for the development of novel treatment to improve the survival of neuroendocrine carcinoma patients.

The treatment for low grade or intermediate grade NETs is different from that of high grade neuroendocrine carcinoma. For non-high grade NETs, several agents, including everolimus, sunitinib and lanreotide, have been shown to improve the progression free survival of advanced GEP and pulmonary NETs^6,9–11^. In addition, chemotherapy with temozolomide alone or in combination with capecitabine has also been shown effective in treating advanced GEP and pulmonary NETs^32–34^. Because in Taiwan the targeted agents for non-pancreatic gastrointestinal tract and pulmonary NETs were approved and reimbursed later than those for pancreatic NETs, it was reasonable for our analysis to show that only pancreatic NETs had an improved overall survival from T2 to T3. However, we can expect that the survival of other gastrointestinal tract and pulmonary NETs will be improved in the near future. In addition to the common sites of NETs, NETs of some other less common sites had poor survival rates, including NETs of head and neck, female genital organs, liver, esophagus, biliary tract and prostate, with 5-year overall survival rates less than 40% (Table 3). Fewer studies have been conducted in these sites of NETs probably due to the rarity and difficulty for data and sample collection, leading to a paucity of information to improve their survival.

This study has a few limitations. First, we did not have complete data on tumor grade, because the tumor grade data were not consistently reported to the TCR by the hospitals, especially for rare cancers such as NETs. Second, the TCR does not routinely collect data on Ki67 and somatostatin receptors and therefore data on these markers were not available.

In summary, we showed a rapid increase in the incidence of NETs in Taiwan, which is probably due to multiple factors, including increased awareness by the physicians, diagnostic improvement, and possible increase of unidentified risk factors. The overall survival of pancreatic NETs improved after introduction of targeted therapies whereas the survival improvement of other gastrointestinal tract and pulmonary NETs awaits evaluation in the near future. It is important to identify the etiologies and understand the pathogenesis of NETs to prevent the occurrence of NETs. In addition, development of novel treatment agents is crucial to improve the survival of NETs.

## Methods

The content and the execution of the current study were approved by The Research Ethics Committee of the National Health Research Institutes (NHRI), Taiwan. All methods were carried out in accordance with the guidelines and regulations of NHRI. Because the analysis was based on de-identified secondary data, individual consent was not required. The consent waiver was approved by the Research Ethics Committee of the NHRI.

Data used in the current analysis were based on information contained in the TCR and the Death Registry Database located at the Center for Medical Informatics and Statistics under the supervision of the Ministry of Health and Welfare. Approximately 97% of all cancer diagnoses are capture by the TCR, which began to monitor the incidence and mortality rates of cancers in Taiwan in 1979. Detailed information about the quality of the TCR was described previously^3^.

The morphology (M) codes of the International Classification of Diseases for Oncology, Field trial edition (ICD-O-FT) or the International Classification of Diseases for Oncology, third edition (ICD-O-3) were used to identify NETs, including 8240 (carcinoid tumor), 8241 (enterochromaffin cell carcinoid), 8242 (enterochromaffin-like cell tumors), 8243 (goblet cell carcinoid), 8244 (composite carcinoid), 8245 (adenocarcinoid), 8246 (neuroendocrine carcinoma), 8249 (atypical carcinoid), 8013 (large cell neuroendocrine carcinoma), and 8574 (adenocarcinoma with neuroendocrine differentiation) as described previously^3^.

The annual populations from 1996 to 2015 reported by the Directorate-General of Budget, Accounting, and Statistics of Taiwan (http://www.dgbas.gov.tw) were used as denominators to calculate the crude annual incidence rates. Age-standardized incidence rates were calculated by direct standardization to the age distribution of the 2000 WHO standard population. To assess any shift in the incidence trends of NETs overall and by sites, we calculated the annual percentage change (APC) using linear regression: log(rate_y_) = b_0_ + b_1_y, with log(rate_y_) = natural log of incidence rate in year y. The APC = (e^b1^ − 1) × 100.

To evaluate the overall survival of NETs, data on vital status and the date of death were ascertained from the Death Registry Database. Life-table method was used to estimate the 1-, 3-, 5-, and 10-year overall survival of NETs overall, by sex, by site, and by the time period of diagnosis (T1: from 1996–2003, T2: from 2004–2009, and T3: from 2010–2015). The risk of NETs death was evaluated by performing Cox proportional hazards regression analysis to calculate the hazard ratio (HR) and 95% confidence interval (CI) associated with age, sex, site, and the time period of diagnosis.

### Ethics approval and consent to participate

The content and the execution of the current study were approved by The Research Ethics Committee of the National Health Research Institutes, Taiwan.

### Consent for publication

Because the analysis was based on de-identified secondary data, individual consent was not required.

## Supplementary Information

Supplementary Table 1.

Supplementary Table 2.

Supplementary Table 3.

## Data Availability

The data that support the findings of this study are available from the Center for Medical Informatics and Statistics under the supervision of the Ministry of Health and Welfare, but restrictions apply to the availability of these data, which were used under license for the current study, and so are not publicly available.
