# Supplementary material for: An updated analysis of the epidemiologic trends of neuroendocrine tumors in Taiwan
Source: Sci Rep. 2021 Apr 12;11:7881. doi: 10.1038/s41598-021-86839-2 (PMC8041887; doi:10.1038/s41598-021-86839-2)
Supplement: Supplementary file 1 — Supplementary Table 1. [file 41598_2021_86839_MOESM1_ESM.doc]

**An updated analysis of the epidemiologic trends of neuroendocrine tumors in Taiwan**

Jeffrey S. Chang^1^, Li-Tzong Chen^1,2,3^, Yan-Shen Shan^4,5^, Pei-Yi Chu^1,6,7^, Chia-Rong Tsai^1^, Hui-Jen Tsai^1,2,3^

**Supplementary Table 1.** The annual incidence (cases per 100,000) of NETs by the site and sex from 1996 to 2015

|  | **1996** | **1997** | **1998** | **1999** | **2000** | **2001** | **2002** | **2003** | **2004** | **2005** | **2006** | **2007** | **2008** | **2009** | **2010** | **2011** | **2012** | **2013** | **2014** | **2015** | **APC** | **P value** |
| --- | --- | --- | --- | --- | --- | --- | --- | --- | --- | --- | --- | --- | --- | --- | --- | --- | --- | --- | --- | --- | --- | --- |
| **ALL NETs** | **0.244** | **0.289** | **0.337** | **0.388** | **0.455** | **0.364** | **0.618** | **0.597** | **0.739** | **0.982** | **1.049** | **1.298** | **1.306** | **1.583** | **1.789** | **2.080** | **2.745** | **2.928** | **3.247** | **3.162** | 15.44 | <0.0001 |
| Men | 0.287 | 0.343 | 0.384 | 0.496 | 0.590 | 0.437 | 0.806 | 0.730 | 0.817 | 1.205 | 1.307 | 1.545 | 1.734 | 1.921 | 2.183 | 2.422 | 3.261 | 3.497 | 3.764 | 3.612 | 15.26 | <0.0001 |
| Women | 0.196 | 0.243 | 0.279 | 0.269 | 0.314 | 0.292 | 0.427 | 0.463 | 0.657 | 0.756 | 0.796 | 1.057 | 0.895 | 1.268 | 1.423 | 1.773 | 2.267 | 2.413 | 2.770 | 2.748 | 15.99 | <0.0001 |
| **Primary site** | | | | | | | | | | | | | | | | | | | | | | |
| **Rectum** | **0.059** | **0.091** | **0.111** | **0.087** | **0.108** | **0.082** | **0.160** | **0.088** | **0.181** | **0.222** | **0.284** | **0.373** | **0.350** | **0.455** | **0.492** | **0.539** | **0.896** | **1.002** | **1.110** | **1.149** | 17.34 | <0.0001 |
| Men | 0.061 | 0.105 | 0.145 | 0.096 | 0.133 | 0.083 | 0.203 | 0.116 | 0.262 | 0.261 | 0.293 | 0.468 | 0.441 | 0.546 | 0.601 | 0.674 | 1.126 | 1.229 | 1.335 | 1.400 | 17.81 | <0.0001 |
| Women | 0.056 | 0.075 | 0.074 | 0.077 | 0.083 | 0.082 | 0.117 | 0.059 | 0.100 | 0.181 | 0.279 | 0.277 | 0.263 | 0.368 | 0.387 | 0.413 | 0.672 | 0.786 | 0.892 | 0.907 | 17.03 | <0.0001 |
| **Lung and bronchus** | **0.042** | **0.033** | **0.074** | **0.090** | **0.121** | **0.081** | **0.170** | **0.140** | **0.131** | **0.201** | **0.211** | **0.229** | **0.214** | **0.317** | **0.315** | **0.382** | **0.414** | **0.437** | **0.470** | **0.399** | 13.42 | <0.0001 |
| Men | 0.053 | 0.038 | 0.090 | 0.101 | 0.163 | 0.126 | 0.262 | 0.232 | 0.159 | 0.322 | 0.311 | 0.289 | 0.350 | 0.485 | 0.510 | 0.523 | 0.627 | 0.637 | 0.649 | 0.536 | 14.39 | <0.0001 |
| Women | 0.032 | 0.030 | 0.055 | 0.074 | 0.075 | 0.034 | 0.078 | 0.047 | 0.103 | 0.079 | 0.112 | 0.171 | 0.084 | 0.165 | 0.131 | 0.254 | 0.221 | 0.257 | 0.308 | 0.281 | 12.26 | <0.0001 |
| **Pancreas** | **0.017** | **0.004** | **0.014** | **0.004** | **0.009** | **0.025** | **0.017** | **0.029** | **0.056** | **0.048** | **0.082** | **0.097** | **0.112** | **0.116** | **0.170** | **0.255** | **0.400** | **0.447** | **0.404** | **0.446** | 28.04 | <0.0001 |
| Men | 0.008 | 0.000 | 0.008 | 0.000 | 0.018 | 0.033 | 0.016 | 0.048 | 0.026 | 0.036 | 0.077 | 0.101 | 0.119 | 0.065 | 0.204 | 0.280 | 0.454 | 0.421 | 0.379 | 0.516 | 26.87 | <0.0001 |
| Women | 0.027 | 0.009 | 0.020 | 0.009 | 0.000 | 0.016 | 0.018 | 0.009 | 0.085 | 0.060 | 0.087 | 0.095 | 0.104 | 0.165 | 0.140 | 0.234 | 0.348 | 0.473 | 0.430 | 0.379 | 24.0 | <0.0001 |
| **Stomach** | **0.021** | **0.034** | **0.010** | **0.031** | **0.026** | **0.028** | **0.047** | **0.043** | **0.038** | **0.076** | **0.077** | **0.092** | **0.099** | **0.137** | **0.149** | **0.133** | **0.166** | **0.221** | **0.265** | **0.242** | 15.96 | <0.0001 |
| Men | 0.030 | 0.063 | 0.011 | 0.054 | 0.033 | 0.014 | 0.077 | 0.053 | 0.046 | 0.098 | 0.116 | 0.106 | 0.137 | 0.157 | 0.210 | 0.173 | 0.180 | 0.217 | 0.349 | 0.265 | 15.0 | <0.0001 |
| Women | 0.011 | 0.010 | 0.010 | 0.007 | 0.018 | 0.043 | 0.017 | 0.033 | 0.030 | 0.052 | 0.036 | 0.077 | 0.064 | 0.118 | 0.092 | 0.097 | 0.152 | 0.230 | 0.192 | 0.225 | 19.72 | <0.0001 |
| **Colon** | **0.010** | **0.028** | **0.021** | **0.025** | **0.017** | **0.022** | **0.019** | **0.022** | **0.043** | **0.076** | **0.058** | **0.084** | **0.090** | **0.080** | **0.100** | **0.106** | **0.172** | **0.141** | **0.248** | **0.138** | 15.63 | <0.0001 |
| Men | 0.000 | 0.039 | 0.017 | 0.030 | 0.007 | 0.027 | 0.013 | 0.014 | 0.060 | 0.124 | 0.076 | 0.099 | 0.105 | 0.099 | 0.119 | 0.114 | 0.168 | 0.191 | 0.334 | 0.166 | 17.46 | <0.0001 |
| Women | 0.020 | 0.016 | 0.025 | 0.019 | 0.027 | 0.017 | 0.025 | 0.028 | 0.025 | 0.028 | 0.041 | 0.069 | 0.074 | 0.063 | 0.082 | 0.098 | 0.174 | 0.094 | 0.166 | 0.109 | 13.1 | <0.0001 |
| **Small intestine** | **0.021** | **0.014** | **0.028** | **0.014** | **0.018** | **0.023** | **0.025** | **0.046** | **0.048** | **0.053** | **0.048** | **0.068** | **0.049** | **0.109** | **0.090** | **0.055** | **0.108** | **0.128** | **0.150** | **0.119** | 12.68 | <0.0001 |
| Men | 0.041 | 0.019 | 0.041 | 0.029 | 0.035 | 0.021 | 0.041 | 0.048 | 0.055 | 0.047 | 0.072 | 0.103 | 0.087 | 0.133 | 0.082 | 0.069 | 0.133 | 0.185 | 0.182 | 0.134 | 10.81 | <0.0001 |
| Women | 0.000 | 0.009 | 0.012 | 0.000 | 0.000 | 0.023 | 0.008 | 0.044 | 0.041 | 0.060 | 0.024 | 0.034 | 0.013 | 0.084 | 0.098 | 0.042 | 0.084 | 0.075 | 0.120 | 0.104 | 13.99 | 0.0001 |
| **Appendix** | **0.015** | **0.004** | **0.010** | **0.013** | **0.026** | **0.009** | **0.018** | **0.012** | **0.051** | **0.037** | **0.036** | **0.041** | **0.047** | **0.033** | **0.055** | **0.083** | **0.135** | **0.083** | **0.135** | **0.182** | 17.0 | <0.0001 |
| Men | 0.028 | 0.000 | 0.008 | 0.025 | 0.042 | 0.018 | 0.028 | 0.011 | 0.056 | 0.033 | 0.051 | 0.033 | 0.070 | 0.034 | 0.050 | 0.072 | 0.134 | 0.109 | 0.157 | 0.174 | 12.82 | <0.0001 |
| Women | 0.000 | 0.009 | 0.010 | 0.000 | 0.007 | 0.000 | 0.008 | 0.015 | 0.046 | 0.043 | 0.022 | 0.048 | 0.024 | 0.032 | 0.062 | 0.097 | 0.141 | 0.056 | 0.111 | 0.192 | 18.49 | <0.0001 |
| **Thymus and mediastinum** | **0.008** | **0.019** | **0.017** | **0.024** | **0.023** | **0.013** | **0.031** | **0.033** | **0.012** | **0.038** | **0.043** | **0.038** | **0.048** | **0.056** | **0.054** | **0.063** | **0.045** | **0.064** | **0.054** | **0.045** | 8.8 | <0.0001 |
| Men | 0.015 | 0.028 | 0.023 | 0.048 | 0.045 | 0.025 | 0.045 | 0.039 | 0.006 | 0.058 | 0.058 | 0.062 | 0.057 | 0.082 | 0.080 | 0.097 | 0.078 | 0.114 | 0.074 | 0.064 | 8.33 | 0.001 |
| Women | 0.000 | 0.010 | 0.011 | 0.000 | 0.000 | 0.000 | 0.016 | 0.027 | 0.016 | 0.016 | 0.027 | 0.015 | 0.039 | 0.030 | 0.032 | 0.031 | 0.014 | 0.016 | 0.036 | 0.026 | 4.82 | 0.02 |
| **Head and neck** | **0.009** | **0.036** | **0.017** | **0.008** | **0.015** | **0.018** | **0.028** | **0.025** | **0.023** | **0.053** | **0.020** | **0.051** | **0.057** | **0.042** | **0.061** | **0.057** | **0.054** | **0.058** | **0.026** | **0.036** | 7.65 | 0.0006 |
| Men | 0.017 | 0.044 | 0.024 | 0.015 | 0.022 | 0.028 | 0.022 | 0.027 | 0.022 | 0.091 | 0.033 | 0.068 | 0.087 | 0.081 | 0.111 | 0.052 | 0.082 | 0.100 | 0.035 | 0.050 | 7.75 | 0.0009 |
| Women | 0.000 | 0.030 | 0.008 | 0.000 | 0.007 | 0.008 | 0.034 | 0.023 | 0.023 | 0.015 | 0.007 | 0.033 | 0.027 | 0.005 | 0.013 | 0.063 | 0.027 | 0.018 | 0.018 | 0.021 | 2.88 | 0.36 |
| **Female genital** | **0.008** | **0.004** | **0.014** | **0.008** | **0.017** | **0.008** | **0.023** | **0.032** | **0.014** | **0.030** | **0.022** | **0.036** | **0.036** | **0.043** | **0.045** | **0.063** | **0.057** | **0.063** | **0.068** | **0.050** | - | - |
| Women | 0.018 | 0.008 | 0.029 | 0.017 | 0.035 | 0.017 | 0.047 | 0.065 | 0.028 | 0.060 | 0.044 | 0.070 | 0.071 | 0.085 | 0.089 | 0.125 | 0.113 | 0.125 | 0.132 | 0.097 | 12.96 | <0.0001 |
| **Breast** | **0.000** | **0.000** | **0.000** | **0.000** | **0.000** | **0.004** | **0.000** | **0.000** | **0.022** | **0.019** | **0.031** | **0.036** | **0.014** | **0.018** | **0.044** | **0.034** | **0.053** | **0.063** | **0.070** | **0.065** | - | - |
| Men | 0.000 | 0.000 | 0.000 | 0.000 | 0.000 | 0.000 | 0.000 | 0.000 | 0.000 | 0.000 | 0.008 | 0.000 | 0.000 | 0.000 | 0.000 | 0.000 | 0.000 | 0.000 | 0.000 | 0.000 | - | - |
| Women | 0.000 | 0.000 | 0.000 | 0.000 | 0.000 | 0.007 | 0.000 | 0.000 | 0.043 | 0.038 | 0.053 | 0.071 | 0.028 | 0.036 | 0.083 | 0.065 | 0.103 | 0.122 | 0.138 | 0.124 | 18.05 | 0.0002 |
| **Liver** | **0.008** | **0.004** | **0.004** | **0.009** | **0.005** | **0.005** | **0.008** | **0.022** | **0.015** | **0.030** | **0.000** | **0.011** | **0.031** | **0.019** | **0.027** | **0.024** | **0.034** | **0.031** | **0.033** | **0.030** | 11.74 | <0.0001 |
| Men | 0.008 | 0.000 | 0.000 | 0.018 | 0.010 | 0.009 | 0.017 | 0.035 | 0.010 | 0.013 | 0.000 | 0.006 | 0.040 | 0.039 | 0.033 | 0.043 | 0.032 | 0.047 | 0.036 | 0.038 | 9.17 | 0.001 |
| Women | 0.008 | 0.008 | 0.009 | 0.000 | 0.000 | 0.000 | 0.000 | 0.008 | 0.022 | 0.047 | 0.000 | 0.014 | 0.023 | 0.000 | 0.020 | 0.006 | 0.035 | 0.016 | 0.030 | 0.024 | 5.45 | 0.05 |
| **Esophagus** | **0.000** | **0.000** | **0.000** | **0.000** | **0.004** | **0.006** | **0.003** | **0.008** | **0.013** | **0.007** | **0.015** | **0.021** | **0.010** | **0.017** | **0.016** | **0.030** | **0.039** | **0.010** | **0.029** | **0.038** | 14.38 | <0.0001 |
| Men | 0.000 | 0.000 | 0.000 | 0.000 | 0.007 | 0.011 | 0.007 | 0.016 | 0.025 | 0.013 | 0.023 | 0.042 | 0.021 | 0.026 | 0.027 | 0.061 | 0.068 | 0.020 | 0.057 | 0.080 | 14.5 | <0.0001 |
| Women | 0.000 | 0.000 | 0.000 | 0.000 | 0.000 | 0.000 | 0.000 | 0.000 | 0.000 | 0.000 | 0.007 | 0.000 | 0.000 | 0.008 | 0.006 | 0.000 | 0.012 | 0.000 | 0.004 | 0.000 | -3.43 | 0.67 |
| **Female gonads** | **0.004** | **0.000** | **0.004** | **0.010** | **0.000** | **0.005** | **0.009** | **0.004** | **0.011** | **0.004** | **0.011** | **0.007** | **0.010** | **0.015** | **0.024** | **0.030** | **0.024** | **0.032** | **0.006** | **0.023** | - | - |
| Women | 0.010 | 0.000 | 0.008 | 0.021 | 0.000 | 0.009 | 0.018 | 0.007 | 0.023 | 0.008 | 0.023 | 0.013 | 0.019 | 0.030 | 0.048 | 0.059 | 0.046 | 0.063 | 0.013 | 0.045 | 9.2 | 0.002 |
| **Urinary tract** | **0.008** | **0.000** | **0.000** | **0.006** | **0.005** | **0.000** | **0.000** | **0.004** | **0.005** | **0.011** | **0.011** | **0.022** | **0.007** | **0.019** | **0.012** | **0.024** | **0.017** | **0.011** | **0.023** | **0.030** | 8.85 | 0.0009 |
| Men | 0.000 | 0.000 | 0.000 | 0.014 | 0.000 | 0.000 | 0.000 | 0.000 | 0.000 | 0.007 | 0.007 | 0.029 | 0.014 | 0.025 | 0.012 | 0.044 | 0.031 | 0.020 | 0.030 | 0.020 | 7.04 | 0.08 |
| Women | 0.013 | 0.000 | 0.000 | 0.000 | 0.010 | 0.000 | 0.000 | 0.007 | 0.009 | 0.015 | 0.014 | 0.015 | 0.000 | 0.014 | 0.011 | 0.005 | 0.005 | 0.005 | 0.018 | 0.037 | 0.57 | 0.85 |
| **Biliary tract** | **0.000** | **0.000** | **0.000** | **0.000** | **0.006** | **0.008** | **0.000** | **0.004** | **0.004** | **0.006** | **0.000** | **0.003** | **0.014** | **0.013** | **0.010** | **0.011** | **0.020** | **0.027** | **0.023** | **0.019** | 11.98 | 0.001 |
| Men | 0.000 | 0.000 | 0.000 | 0.000 | 0.011 | 0.007 | 0.000 | 0.000 | 0.000 | 0.000 | 0.000 | 0.000 | 0.014 | 0.015 | 0.006 | 0.000 | 0.013 | 0.046 | 0.007 | 0.008 | 2.08 | 0.65 |
| Women | 0.000 | 0.000 | 0.000 | 0.000 | 0.000 | 0.008 | 0.000 | 0.008 | 0.008 | 0.014 | 0.000 | 0.006 | 0.013 | 0.012 | 0.013 | 0.021 | 0.026 | 0.011 | 0.038 | 0.029 | 10.41 | 0.002 |
| **Prostate** | **0.000** | **0.000** | **0.000** | **0.000** | **0.000** | **0.000** | **0.007** | **0.004** | **0.000** | **0.007** | **0.011** | **0.013** | **0.000** | **0.008** | **0.003** | **0.020** | **0.007** | **0.015** | **0.019** | **0.016** | - | - |
| Men | 0.000 | 0.000 | 0.000 | 0.000 | 0.000 | 0.000 | 0.014 | 0.009 | 0.000 | 0.014 | 0.021 | 0.026 | 0.000 | 0.016 | 0.006 | 0.042 | 0.014 | 0.032 | 0.040 | 0.033 | 7.89 | 0.07 |
| **Others** | **0.014** | **0.018** | **0.012** | **0.056** | **0.057** | **0.030** | **0.051** | **0.082** | **0.073** | **0.064** | **0.091** | **0.078** | **0.121** | **0.085** | **0.122** | **0.170** | **0.106** | **0.094** | **0.115** | **0.138** | 11.51 | <0.0001 |
| Men | 0.027 | 0.008 | 0.016 | 0.065 | 0.064 | 0.034 | 0.061 | 0.080 | 0.092 | 0.088 | 0.162 | 0.112 | 0.193 | 0.118 | 0.130 | 0.178 | 0.119 | 0.130 | 0.101 | 0.129 | 11.57 | <0.0001 |
| Women | 0.000 | 0.029 | 0.008 | 0.045 | 0.051 | 0.026 | 0.040 | 0.083 | 0.055 | 0.040 | 0.021 | 0.047 | 0.049 | 0.053 | 0.115 | 0.163 | 0.094 | 0.064 | 0.126 | 0.146 | 10.08 | 0.0003 |
