# Supplementary material for: An updated analysis of the epidemiologic trends of neuroendocrine tumors in Taiwan
Source: Sci Rep. 2021 Apr 12;11:7881. doi: 10.1038/s41598-021-86839-2 (PMC8041887; doi:10.1038/s41598-021-86839-2)
Supplement: Supplementary file 2 — Supplementary Table 2. [file 41598_2021_86839_MOESM2_ESM.doc]

**An updated analysis of the epidemiologic trends of neuroendocrine tumors in Taiwan**

Jeffrey S. Chang^1^, Li-Tzong Chen^1,2,3^, Yan-Shen Shan^4,5^, Pei-Yi Chu^1,6,7^, Chia-Rong Tsai^1^, Hui-Jen Tsai^1,2,3^

**Supplementary Table 2**. The M code distribution overall and by sex in NETs of the 6 most common sites in the three time periods.

|  | Rectum | | | | | | | | |
| --- | --- | --- | --- | --- | --- | --- | --- | --- | --- |
|  | T1 (1996-2003) | | | T2 (2004-2009) | | | T3 (2010-2015) | | |
|  | All | Men | Women | All | Men | Women | All | Men | Women |
| M code | N (%) | N (%) | N (%) | N (%) | N (%) | N (%) | N (%) | N (%) | N (%) |
| 8240 (carcinoid) | 173(94.0) | 104(93.7) | 69(94.5) | 445(86.6) | 263(84.8) | 182(89.2) | 1305(81.4) | 783(81.3) | 522(81.6) |
| 8246 (neuroendocrine carcinoma) | 9(4.9) | 5(4.5) | 4(5.5) | 48(9.3) | 33(10.6) | 15(7.4) | 183(11.4) | 111(11.5) | 72(11.3) |
| others | 2(1.1) | 2(1.8) | 0(0) | 21(4.1) | 14(4.5) | 7(3.4) | 115(7.2) | 69(7.2) | 46(7.2) |

|  | Lung and bronchus | | | | | | | | |
| --- | --- | --- | --- | --- | --- | --- | --- | --- | --- |
|  | T1 (1996-2003) | | | T2 (2004-2009) | | | T3 (2010-2015) | | |
|  | All | Men | Women | All | Men | Women | All | Men | Women |
| M code | N (%) | N (%) | N (%) | N (%) | N (%) | N (%) | N (%) | N (%) | N (%) |
| 8240 (carcinoid) | 77(44.5) | 45(35.7) | 32(68.1) | 84(23.1) | 30(11.2) | 54(55.7) | 154(19.3) | 56(10.1) | 98(40) |
| 8249 (atypical carcinoid) | 5(2.9) | 2(1.6) | 3(6.4) | 32(8.8) | 25(9.4) | 7(7.2) | 62(7.8) | 25(4.5) | 37(15.1) |
| 8246 (neuroendocrine carcinoma) | 65(37.6) | 56(44.4) | 9(19.1) | 122(33.5) | 96(36.0) | 26(26.7) | 231(28.9) | 182(32.9) | 49(20) |
| 8013 (large cell neuroendocrine carcinoma) | 24(13.9) | 21(16.7) | 3(6.4) | 110(30.2) | 100(37.5) | 10(10.3) | 273(34.2) | 229(41.3) | 44(18.0) |
| others | 2(1.2) | 2(1.6) | 0(0) | 16(4.4) | 16(6.0) | 0(0) | 79(9.9) | 62(11.2) | 17(6.9) |

|  | Pancreas | | | | | | | | |
| --- | --- | --- | --- | --- | --- | --- | --- | --- | --- |
|  | T1 (1996-2003) | | | T2 (2004-2009) | | | T3 (2010-2015) | | |
|  | All | Men | Women | All | Men | Women | All | Men | Women |
| M code | N (%) | N (%) | N (%) | N (%) | N (%) | N (%) | N (%) | N (%) | N (%) |
| 8240 (carcinoid) | 7(25) | 4(25) | 3(25) | 6(4.3) | 2(3.4) | 4(4.9) | 229(34.5) | 103(29.9) | 126(39.6) |
| 8246 (neuroendocrine carcinoma) | 20(71.4) | 11(68.8) | 9(75) | 131(93.6) | 53(91.4) | 78(95.1) | 266(40.1) | 152(44.1) | 114(35.8) |
| others | 1(3.6) | 1(6.3) | 0(0) | 3(2.1) | 3(5.2) | 0(0) | 168(25.3) | 90(26.1) | 78(24.5) |

|  | Stomach | | | | | | | | |
| --- | --- | --- | --- | --- | --- | --- | --- | --- | --- |
|  | T1 (1996-2003) | | | T2 (2004-2009) | | | T3 (2010-2015) | | |
|  | All | Men | Women | All | Men | Women | All | Men | Women |
| M code | N (%) | N (%) | N (%) | N (%) | N (%) | N (%) | N (%) | N (%) | N (%) |
| 8240 (carcinoid) | 39(73.6) | 23(60.9) | 16(94.1) | 68(45.6) | 31(32.3) | 37(69.8) | 157(39.7) | 58(25.2) | 99(60) |
| 8246 (neuroendocrine carcinoma) | 11(20.8) | 10(27.8) | 1(5.9) | 49(32.9) | 38(39.6) | 11(20.8) | 109(27.6) | 78(33.9) | 31(18.8) |
| others | 3(5.7) | 3(8.3) | 0(0) | 32(21.5) | 27(28.1) | 5(9.4) | 129(32.7) | 94(40.9) | 35(21.2) |

|  | Colon | | | | | | | | |
| --- | --- | --- | --- | --- | --- | --- | --- | --- | --- |
|  | T1 (1996-2003) | | | T2 (2004-2009) | | | T3 (2010-2015) | | |
|  | All | Men | Women | All | Men | Women | All | Men | Women |
| M code | N (%) | N (%) | N (%) | N (%) | N (%) | N (%) | N (%) | N (%) | N (%) |
| 8240 (carcinoid) | 30(78.9) | 17(94.4) | 13(65) | 49(41.2) | 32(42.7) | 17(38.6) | 106(36.3) | 68(40) | 38(31.1) |
| 8246 (neuroendocrine carcinoma) | 7(18.4) | 1(5.6) | 6(30) | 35(29.4) | 21(28) | 14(31.8) | 68(23.3) | 46(27.1) | 22(18.0) |
| others | 1(2.6) | 0(0) | 1(5) | 35(29.4) | 22(29.3) | 13(29.5) | 118(40.4) | 56(32.9) | 62(50.8) |

|  | Small intestine | | | | | | | | |
| --- | --- | --- | --- | --- | --- | --- | --- | --- | --- |
|  | T1 (1996-2003) | | | T2 (2004-2009) | | | T3 (2010-2015) | | |
|  | All | Men | Women | All | Men | Women | All | Men | Women |
| M code | N (%) | N (%) | N (%) | N (%) | N (%) | N (%) | N (%) | N (%) | N (%) |
| 8240 (carcinoid) | 34(79.1) | 26(81.3) | 8(72.7) | 62(59.0) | 39(56.5) | 23(63.9) | 130(59.6) | 74(58.7) | 56(60.9) |
| 8246 (neuroendocrine carcinoma) | 7(16.3) | 5(15.6) | 2(18.2) | 37(35.2) | 25(36.2) | 12(33.3) | 55(25.2) | 30(23.8) | 25(27.2) |
| others | 2(4.7) | 1(3.1) | 1(9.1) | 6(5.7) | 5(7.2) | 1(2.8) | 33(15.1) | 22(17.5) | 11(12.0) |
