# Supplementary material for: An updated analysis of the epidemiologic trends of neuroendocrine tumors in Taiwan
Source: Sci Rep. 2021 Apr 12;11:7881. doi: 10.1038/s41598-021-86839-2 (PMC8041887; doi:10.1038/s41598-021-86839-2)
Supplement: Supplementary file 3 — Supplementary Table 3. [file 41598_2021_86839_MOESM3_ESM.docx]

**An updated analysis of the epidemiologic trends of neuroendocrine tumors in Taiwan**

Jeffrey S. Chang^1^, Li-Tzong Chen^1,2,3^, Yan-Shen Shan^4,5^, Pei-Yi Chu^1,6,7^, Chia-Rong Tsai^1^, Hui-Jen Tsai^1,2,3^

**Supplementary Table 3.** The 1-, 3-, 5-, and 10-year overall survival rates of NETs in six common sites by sex and diagnosed periods.

T1 (1996-2003)

|  | 1-year | | | 3-year | | | 5-year | | | 10-year | | |
| --- | --- | --- | --- | --- | --- | --- | --- | --- | --- | --- | --- | --- |
|  | All | Men | Women | All | Men | Women | All | Men | Women | All | Men | Women |
| All | 0.751 | 0.699 | 0.839 | 0.591 | 0.533 | 0.688 | 0.523 | 0.459 | 0.632 | 0.416 | 0.353 | 0.523 |
| Primary site | | | | | | | | | | | | |
| Rectum | 0.913 | 0.892 | 0.945 | 0.853 | 0.838 | 0.877 | 0.799 | 0.775 | 0.836 | 0.728 | 0.703 | 0.767 |
| Lung and bronchus | 0.676 | 0.627 | 0.809 | 0.445 | 0.365 | 0.660 | 0.399 | 0.310 | 0.638 | 0.318 | 0.246 | 0.511 |
| Pancreas | 0.821 | 0.750 | 0.917 | 0.643 | 0.500 | 0.833 | 0.429 | 0.250 | 0.667 | 0.286 | 0.188 | 0.417 |
| Stomach | 0.679 | 0.528 | 1.000 | 0.472 | 0.306 | 0.824 | 0.434 | 0.306 | 0.706 | 0.264 | 0.167 | 0.471 |
| Colon | 0.658 | 0.722 | 0.600 | 0.579 | 0.611 | 0.550 | 0.553 | 0.556 | 0.550 | 0.526 | 0.500 | 0.550 |
| Small intestine | 0.674 | 0.625 | 0.818 | 0.535 | 0.500 | 0.636 | 0.465 | 0.406 | 0.636 | 0.326 | 0.281 | 0.455 |

T2 (2004-2009)

|  | 1-year | | | 3-year | | | 5-year | | | 10-year | | |
| --- | --- | --- | --- | --- | --- | --- | --- | --- | --- | --- | --- | --- |
|  | All | Men | Women | All | Men | Women | All | Men | Women | All | Men | Women |
| All | 0.716 | 0.658 | 0.807 | 0.565 | 0.502 | 0.664 | 0.501 | 0.440 | 0.596 | 0.421 | 0.365 | 0.510 |
| Primary site | | | | | | | | | | | | |
| Rectum | 0.930 | 0.910 | 0.961 | 0.877 | 0.858 | 0.907 | 0.841 | 0.819 | 0.873 | 0.755 | 0.737 | 0.783 |
| Lung and bronchus | 0.534 | 0.444 | 0.784 | 0.333 | 0.218 | 0.650 | 0.306 | 0.196 | 0.608 | 0.255 | 0.154 | 0.523 |
| Pancreas | 0.614 | 0.569 | 0.646 | 0.436 | 0.414 | 0.451 | 0.329 | 0.293 | 0.354 | 0.126 | 0.120 | 0.126 |
| Stomach | 0.671 | 0.594 | 0.811 | 0.503 | 0.406 | 0.679 | 0.483 | 0.396 | 0.642 | 0.421 | 0.323 | 0.600 |
| Colon | 0.630 | 0.653 | 0.591 | 0.504 | 0.507 | 0.500 | 0.412 | 0.427 | 0.386 | 0.382 | 0.397 | 0.357 |
| Small intestine | 0.781 | 0.783 | 0.778 | 0.600 | 0.551 | 0.694 | 0.495 | 0.420 | 0.639 | 0.423 | 0.339 | 0.577 |

T3 (2010-2015)

|  | 1-year | | | 3-year | | | 5-year | | |
| --- | --- | --- | --- | --- | --- | --- | --- | --- | --- |
|  | All | Men | Women | All | Men | Women | All | Men | Women |
| All | 0.755 | 0.711 | 0.813 | 0.630 | 0.579 | 0.699 | 0.572 | 0.523 | 0.639 |
| Primary site | | | | | | | | | |
| Rectum | 0.949 | 0.944 | 0.956 | 0.909 | 0.902 | 0.919 | 0.879 | 0.866 | 0.899 |
| Lung and bronchus | 0.522 | 0.419 | 0.755 | 0.361 | 0.245 | 0.630 | 0.316 | 0.208 | 0.565 |
| Pancreas | 0.748 | 0.707 | 0.793 | 0.616 | 0.575 | 0.661 | 0.547 | 0.495 | 0.610 |
| Stomach | 0.668 | 0.578 | 0.794 | 0.555 | 0.439 | 0.718 | 0.482 | 0.360 | 0.654 |
| Colon | 0.658 | 0.647 | 0.672 | 0.496 | 0.507 | 0.481 | 0.471 | 0.507 | 0.439 |
| Small intestine | 0.798 | 0.825 | 0.761 | 0.662 | 0.642 | 0.692 | 0.593 | 0.562 | 0.637 |
